# Unmasking the Placenta–Heart Axis: A Comprehensive Review of Placental Abnormalities in Congenital Heart Disease

**DOI:** 10.3390/diagnostics15172159

**Published:** 2025-08-26

**Authors:** Angeliki Gerede, Ilias Orgianelis, Sofoklis Stavros, Anastasios Potiris, Eirini Drakaki, Ioannis Tsimpoukis, Panagiota Papasozomenou, Ekaterini Domali, Nikolaos Nikolettos, Christos Chatzakis, Makarios Eleftheriades

**Affiliations:** 1 Department of Obstetrics and Gynecology, Democritus University of Thrace, 691 00 Alexandroupolis, Greece; nnikolet@med.duth.gr; 2 School of Medicine, Democritus University of Thrace, 691 00 Alexandroupolis, Greece; eliasnelis@gmail.com; 3 Third Department of Obstetrics and Gynecology, University General Hospital “ATTIKON,” Medical School, National and Kapodistrian University of Athens, 124 62 Athens, Greece; apotiris@med.uoa.gr (A.P.); giannistsimpoukis94@gmail.com (I.T.); 4 First Department of Obstetrics and Gynecology, Alexandra Hospital, Medical School, National and Kapodistrian University of Athens, 115 28 Athens, Greece; eirinidrak@med.uoa.gr (E.D.); kdomali@yahoo.fr (E.D.); 5 Midwifery Department, Health Sciences School, International Hellenic University, 574 00 Thessaloniki, Greece; papasozomenou248@gmail.com; 6 Second Department of Obstetrics and Gynecology, Medical School, Aristotle University of Thessaloniki, 541 24 Thessaloniki, Greece; cchatzakis@gmail.com; 7 Second Department of Obstetrics and Gynecology, University Hospital “Aretaieion”, Medical School, National and Kapodistrian University of Athens, 115 28 Athens, Greece; melefth@med.uoa.gr

**Keywords:** congenital heart disease (CHD), placental imaging, fetal MRI, Doppler ultrasound, placental dysfunction, umbilical venous flow, BOLD imaging

## Abstract

Congenital heart disease (CHD), the most prevalent congenital abnormality, is becoming increasingly acknowledged as a component of a broad fetoplacental pathology. This systematic review summarizes recent imaging-based data linking CHD to quantifiable placental abnormalities. In CHD pregnancies, placenta studies consistently show patterns of altered vascularization, decreased volumetric growth, microstructural heterogeneity, and impaired placental oxygenation. We conducted a thorough literature search from January 2020 to May 2025 to identify studies on placenta function and structure in CHD-affected pregnancies. The included studies primarily utilized MRI and Doppler methods, as well as some modern modalities. Seven studies were included in this review. Placental imaging reveals consistent structural and functional abnormalities in pregnancies affected by congenital heart disease, indicating some possible contribution of the placenta in CHD pathophysiology. Placental imaging may improve outcomes in this susceptible group of pregnancies, improve risk assessment, and direct surveillance when incorporated into prenatal care for congenital heart disease. Future research should concentrate on lesion-specific analysis, longitudinal imaging, and placenta–heart axis-targeting treatment therapies.

## 1. Introduction

Congenital heart disease (CHD) is the most common congenital malformation, affecting approximately 1% of live births globally, and is the sixth most common cause of infant mortality across the world [1,2], but it occurs in a much higher percentage of pregnancies resulting in spontaneous abortion or stillbirth [3]. CHD ranges from minor septal defects to severe, life-threatening malformations requiring immediate postnatal intervention. Despite advances in prenatal screening and neonatal care, CHD remains a leading contributor to perinatal morbidity and mortality [4,5] and is linked to adverse pregnancy outcomes like preterm birth, fetal growth restriction, and hypertensive disorders. [6]. These connections suggest that CHD may not occur in isolation but rather in the context of more generalized fetal pathology.

The placenta, critical for fetal development through endocrine signaling, waste removal, and nutrient transfer, is increasingly studied in relation to fetal health [1,7,8]. With the evolution of imaging methods (most notably Doppler ultrasound, three-dimensional power Doppler, and magnetic resonance imaging (MRI)), noninvasive, in vivo assessment of placental structure and function is increasingly feasible [7,8,9]. A verified noninvasive Doppler-derived technique for directly measuring blood flow from the placenta to the fetus is umbilical venous volume flow (UVVF). Some suggest that ultrasound, the current “gold standard” screening tool during pregnancy, is not suitable for assessing the function or microstructure of the placenta compared to MRI [1,10]. Doppler studies allow the evaluation of uterus–placenta–fetus axis circulation, while placental MRI studies provide insights into detailed morphology, perfusion, and oxygenation with high spatial resolution [11]. These methods are increasingly used in high-risk pregnancies to detect early placental dysfunction [12].

The fetal heart and placenta develop concurrently and share several key developmental pathways [13]. Placental and umbilical cord abnormalities are associated with an increased risk of fetal CHD [14]. Placental dysfunction may exacerbate CHD severity through hypoxia or nutrient imbalances during organogenesis, while abnormal fetal heart hemodynamics can impair placental perfusion, leading to structural and functional anomalies like altered villous branching or reduced vascular density [15,16]. The difficulties in evaluating the organ in utero contribute to our incomplete knowledge of the placenta in CHD. Traditional Doppler indices of placental blood flow, such as the cerebroplacental ratio (CPR) and umbilical artery (UA) flow, can fluctuate significantly and are frequently normal in CHD [17], even though statistically significant abnormal uterine artery Doppler waveforms and reduced placental volumes have been reported in pregnancies with CHD-affected fetuses [18]. This raises the possibility that placental dysfunction contributes to the development or severity of certain CHDs through hypoxia or nutrient supply imbalance during critical periods of organogenesis [19].

On the contrary, there is growing evidence that fetal heart defects may, themselves, impair the fetoplacental vascularization process. The similarities in placental and cardiac developmental pathways support the idea that congenital anomalies in the heart would be associated with abnormalities in the placenta [14]. Abnormal hemodynamics, such as reduced cardiac output or reversed flow patterns, can result in inadequate placental perfusion, leading to secondary structural and functional anomalies [20]. Affected placentas may manifest altered villous branching, vascular infarctions, or reduced vascular density, all of them being findings that are verified by both imaging and histopathological studies [21]. This reciprocity suggests a complex interaction between fetal heart and placental development, rather than a unidirectional cause-and-effect model.

Ultimately, placental imaging in fetuses with congenital heart defects presents a promising, underutilized option for improved prenatal surveillance, aiding in risk assessment, predicting adverse outcomes like stillbirth or fetal growth restriction, and guiding management of high-risk pregnancies. Furthermore, it may also reveal common developmental pathways and possible treatment targets. The aim of this review is to summarize recent data on reported abnormalities in placental imaging studies in CHD pregnancies, highlight pathophysiological links, and assess the placenta’s function as a risk mediator and marker in this intricate clinical landscape as a motivation for future research in the field.

## 2. Materials and Methods

### 2.1. Study Design

A comprehensive literature search was conducted to identify peer-reviewed studies published between 1 January 2020 and April 2025 that examined the relationship between congenital heart defects (CHDs) and placental imaging abnormalities. Six electronic databases were queried: PubMed/Medline, Embase, Scopus, Web of Science, Cochrane Library, and ClinicalTrials.gov. The search strategy combined controlled vocabulary (e.g., MeSH, Emtree) and free-text terms related to “congenital heart disease,” “congenital heart defects,” “fetal heart defects,” “placenta,” “placental imaging,” “placenta insufficiency,” and specific modalities such as “Doppler,” “MRI,” “pBOLD,” “ultrasound,” “vascularization,” and “malperfusion.”

Search filters were applied to restrict results to English-language articles published from 2020 to 2025. Reference lists of included articles and relevant reviews were also screened for additional eligible studies. The full search strings used for each database are provided in Supplementary Table S1.

### 2.2. Eligibility Criteria

Studies were eligible for inclusion if they met the following criteria:

- Population: Human pregnancies with fetuses diagnosed with any form of CHD
- Exposure: Prenatal or postnatal imaging of the placenta (e.g., ultrasound, Doppler, and MRI)
- Outcomes: Documented placental abnormalities (e.g., reduced perfusion, altered vascular indices, and structural changes)
- Study Design: Original research including prospective or retrospective cohorts, case–control studies, cross-sectional analyses, and clinical trials
- Language and Date: Published in English between 2020 and 2025

Exclusion criteria included animal studies, case reports, editorials, conference abstracts without full text, and studies without specific assessment of placental imaging.

### 2.3. Study Selection

Two authors (A.G. and I.O.) independently screened all titles and abstracts using the inclusion criteria. Full-text articles were retrieved for potentially relevant studies. Disagreements at any stage were resolved by consensus or consultation with a third reviewer (S.S.).

Data were extracted independently by two authors (A.G. and I.O.) using a standardized form. The following information was collected from each included study: first author, year of publication, country, study design, sample size, type of CHD, imaging modality used, placental findings, and main conclusions. Where applicable, quantitative results (e.g., Doppler indices and placental volume measurements) were recorded.

## 3. Results

**Figure 1:**
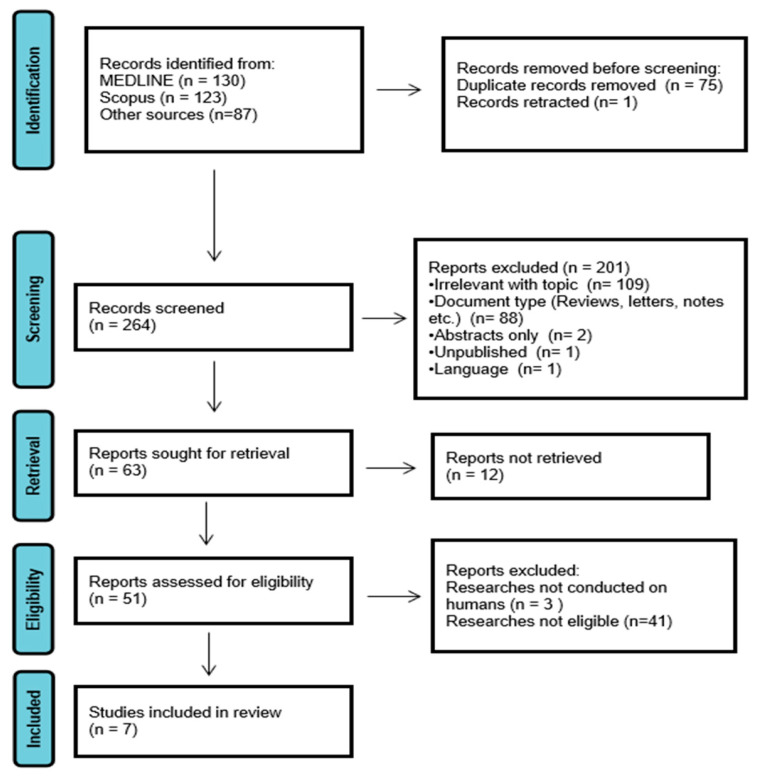
Study selection process.

The initial literature search yielded 340 publications. After deduplicating, 264 studies were deemed potentially relevant and underwent screening. This screening process identified 63 articles, of which the full texts of 51 were successfully retrieved and evaluated for eligibility. All 51 full-text articles were subjected to comprehensive screening, and, ultimately, 7 studies met the predefined inclusion criteria and were included in this comprehensive review. Figure 1 illustrates the study selection process.

### 3.1. Risk of Bias Assessment

The Newcastle–Ottawa Scale (NOS) was utilized to assess the methodological quality of cohort and case–control studies. For cross-sectional studies, a modified NOS or the AXIS tool was employed. This tool is specifically designed for non-randomized studies and evaluates quality based on three broad domains: selection of study groups, comparability of groups, and ascertainment of the exposure or outcome. Each study was independently assessed by two authors (A.P. and C.C.), with any disagreements resolved through discussion with a third author (S.S.). The NOS score ranges from 0 to 9. Studies scoring 8–9 points were classified as low risk of bias, those scoring 6–7 points as moderate risk, and those scoring ≤5 points (none in our dataset) would be considered high risk of bias. Table 1 summarizes the results of the risk of bias assessment.

### 3.2. Reference Groups and Demographic Profile

The seven studies compared the subjects carrying CHD-affected fetuses to demographically matched healthy pregnant individuals. There was no study including a disease–control group apart from fetuses with CHD. The total number of fetuses diagnosed with CHD included across all studies ranged from *n* = 12 to *n* = 71. Table 2 presents the characteristics and the outcomes of included studies.

### 3.3. Placenta Imaging Method and CHD Diagnoses

Across the seven studies, MRI was used to assess placental abnormalities in five studies, and Doppler ultrasound was used in two studies. All of the studies included various types of CHDs, including atrioventricular septal defects (AVSD); congenital heart defects (CHD); double-outlet right ventricle (DORV); hypoplastic left heart syndrome (HLHS); congenitally corrected transposition of great arteries (L-TGA); pulmonary stenosis (PS); tricuspid atresia (TA); transposition of great arteries (TGA); tetralogy of Fallot (TOF); truncus arteriosus (TRUN); ventriculoseptal defect (VSD); coarctation of the aorta (CoA); and right-sided obstructive lesions (RSOL).

### 3.4. MRI Findings

Cromb et al., in their prospective study, employed a rapid whole-uterus multi-echo single-shot gradient-echo EPI sequence, with a total acquisition time under one minute, to assess placental and fetal brain T2* values in fetuses with and without CHD. The study’s fetal participants were scanned at median gestational ages (GAs) of 32.0 weeks for the CHD group and 34.5 weeks for the control group. Of the 51 fetuses with congenital cardiac disease, coarctation of the aorta was the most common diagnosis (*n* = 16), followed by tetralogy of Fallot (*n* = 6), major artery transposition (*n* = 6), and other anomalies. Across the entire cohort, fetal and placental brain T2* values revealed a significant positive association (⍴ = 0.46), persisting independently in both CHD (⍴ = 0.36) and control groups (⍴ = 0.61). Both fetal and placental brain T2* values declined significantly with advancing gestational age (placental T2* ⍴ = –0.65 and fetal brain T2* ⍴ = –0.32), while placental volume increased (⍴ = 0.26). After adjusting for gestational age, CHD fetuses demonstrated significantly lower placental (83 ± 23 ms vs. 97 ± 24 ms) and fetal brain T2* values (202 ± 25 ms vs. 218 ± 26 ms) compared to control groups. The placental volume and thickness showed no significant differences. However, placental texture was lower (more heterogeneous) in CHD pregnancies (median 0.80 vs. 0.84), and morphology scores were higher, indicating bulkier, less uniform placentas. A positive association between placental T2* and texture was observed in the CHD group (⍴ = 0.51) but not in the controls [10].

Furthermore, in a prospective MRI-based study, Cromb et al. compared placental function and structure in 12 pregnancies with CHD and 36 controls using 3T MRI, counting 67 scans in total. After adjusting for gestational and maternal age, mean placental T2* values were notably lower in the CHD group (51.1  ±  9.9 ms) compared to controls (58.1  ±  11.4 ms, *p* = 0.012), suggesting impaired tissue oxygenation. No statistically remarkable differences in placental volume or apparent diffusion coefficient (ADC) were observed between groups. Across all scans, placental volume increased with gestational age (R = 0.50, *p* < 0.0001), while both T2* and ADC declined significantly (R = −0.78 and −0.63, respectively, *p* < 0.0001). Advanced spectral decomposition using InSpect identified seven placental tissue components with distinct microenvironmental signatures. Significant differences in regional component weighting were observed between CHD and control placentas for components 3 and 4 (both pFDR < 0.001). Components 5–7, associated with well-oxygenated lobular cores, decreased across gestation in both groups but more markedly in CHD. Component 7, likely reflecting oxygenated inflow via spiral arteries, declined significantly in CHD placentas (R = −0.89, pFDR < 0.001) [1].

In a prospective MRI study of 108 pregnancies, Jacobwitz et al. compared placental and fetal growth parameters between 53 fetuses with CHD and 55 controls. After adjusting for gestational age and fetal sex, CHD fetuses demonstrated significantly smaller placental volumes across gestation (β = −109.29, SE 28.32; *p*  <  0.001), as well as reduced fetal body volumes (β = −193.60, SE 44.42; *p*  < 0.001). Total brain volume (TBV) was also significantly lower in the CHD cohort (β = −10.87, SE 5.09; *p* = 0.04). Despite these reductions, fetal brain and body growth trajectories remained parallel to those of controls, with no significant differences in growth rates. Subanalysis showed no variation in fetal body volume or fetal-to-placental volume ratio when classified by CHD or lesion type. Interestingly, the fetal-to-placental volume ratio was significantly elevated in the CHD group (β = 0.23, SE 0.10; *p* = 0.02), suggesting disproportionate placental underdevelopment relative to fetal size. No significant association was found between Doppler parameters (MCA-PI, UA-PI, or CPR) and fetal or placental volume metrics [22].

In a prospective cohort study analyzing fetal BOLD MRI data, Rajagopalan et al. evaluated placental and fetal brain oxygenation dynamics in 104 pregnancies, including 30 fetuses with congenital heart defects (CHD). Using intrinsic properties of the BOLD signal as a proxy for vascular tissue integrity and heterogeneity, the authors assessed both placental (pBOLD) and cerebral spatiotemporal variances. While overall spatial and temporal pBOLD variance did not differ significantly between CHD and non-CHD groups after adjusting for GA and sex, CHD status significantly modulated the relationship between GA and both temporal (*p* = 0.028) and spatial (*p* = 0.004) pBOLD variance. Notably, maternal risk factors (MRFs) (e.g., hypertension and diabetes) were independently related to reduced spatiotemporal variance in both placenta and fetal brain BOLD signals. Temporal variance in fetal brain BOLD was notably lower in CHD fetuses compared to the control group (*p* = 0.001), suggesting impaired neurovascular responsiveness. The co-occurrence of CHD and at least one maternal risk factor further reduced placental BOLD temporal variance (*p* = 0.047) [23].

Finally, in their cohort study of 106 pregnancies, including 69 with fetal CHD and 37 controls, Steinweg et al. performed quantitative and qualitative analysis of placental T2* MRI data. Mean gestational age at imaging was approximately 31 weeks. CHD pregnancies, as predicted, scored lower birthweight, head circumference, and gestational age at delivery, alongside a higher incidence of low Apgar scores (*p* = 0.024). Pathology of placentas showed maternal vascular malperfusion (MVM) in two CHD placentas, though not statistically significant. T2* maps in CHD pregnancies revealed diffuse signal loss, accelerated peripheral decay, and increased lobular heterogeneity, particularly in right-sided obstructive lesions (RSOL) and other major cardiac anomalies. Quantitatively, RSOL and related groups showed the lowest mean T2* Z-scores (−2.30 to −2.31), greatest lacunarity (Z = 1.7), and markedly elevated skewness (Z = 4.69) and kurtosis (Z = 3.47), indicating considerable deviation from normal placental oxygenation profiles. Maternal BMI, body position, and placental location did not affect T2* measurements crucially [24].

### 3.5. Doppler Ultrasound Studies

In a prospective study by Ordás et al., 71 fetuses with CHD underwent a series of ultrasound and Doppler evaluation studies between 25 and 40 weeks of gestation to assess fetal growth and placental circulation function. CHD fetuses were classified by expected cerebral oxygenation into two groups, with group I associated with reduced blood and oxygen delivery to the placenta. Compared to controls, CHD fetuses exhibited significantly lower values in the field of fetal growth. On the other hand, Doppler studies revealed higher umbilical artery pulsatility index (UA-PI) (*p*  <  0.001) and lower cerebroplacental ratio (CPR) (*p* = 0.044) in the CHD cohort, with 11.6% showing UA-PI above the 95th centile. Group I demonstrated a greater proportion of abnormal UA-PI (15.4%) and MCA-PI values below the 5th centile (5.4%) (*p*  <  0.001 and *p* = 0.011, respectively). Longitudinally, HC Z-scores declined significantly with the advance of gestation in CHD fetuses, particularly in those with impaired cerebral perfusion (*p*  <  0.001) [26].

Likewise, Josowitz et al., in their study, included 38 fetuses with CHD, predominantly single ventricle (SV) cases, and 36 controls. Median gestational age was higher in cases (23 weeks) than in controls (21 weeks, *p* < 0.001). Fetal weight at scan was significantly greater in CHD cases (629 g [530–756]) and SV fetuses (646 g [528–830]) versus controls (437 g [340–546], both *p* < 0.001). Combined cardiac output (CCO) was similar across groups. Absolute umbilical venous volume flow (UVVF) did not differ significantly (controls: 51 mL/min [38–68]; CHD: 61 mL/min [47–84], *p* = 0.147), but when adjusted to fetal weight (UVVF/Wt), it appeared lower in CHD (96 mL/min/kg [79–115], *p* = 0.007) and SV cases (87 mL/min/kg [74–108], *p* = 0.001) compared to the control group (113 mL/min/kg [98–145]). UVVF as a part of CCO was also reduced in CHD (22% [18–30], *p* = 0.006) and SV fetuses (23% [20–31], *p* = 0.045) relative to controls (30% [24–39]). CHD cases showed higher umbilical artery PI (1.36 [1.27–1.48], *p* = 0.020) and lower uterine artery PI (0.78 [0.66–1.00], *p* = 0.017) compared to controls [25].

## 4. Discussion

It is becoming clear that placental structure and function are impacted in CHD; nonetheless, the start and mechanisms of placental injury and/or maldevelopment remain poorly understood [27]. Recent pathology and imaging advances (particularly fetal MRI and Doppler ultrasound) have illuminated the connection between CHD and placental dysfunction [28]. The seven studies analyzed in this review provide compelling evidence that placental structure, function, and perfusion are consistently altered in pregnancies complicated by fetal CHD. This discussion summarizes these findings in the context of recent existing literature, evaluates methodological limitations, explores pathophysiological mechanisms, and proposes implications for future clinical care and research.

### 4.1. Key Findings of the Present Review

Across the seven studies included in this review, CHD pregnancies exhibit a noticeable pattern of placental abnormality, particularly in oxygenation and microstructural parameters. T2*-weighted MRI data consistently showed reduced placental T2 values * in CHD cases [1,10,24], reflecting impaired oxygenation and vascular heterogeneity.

Volumetric analysis further supports placental compromise. Josowitz et al. demonstrated significantly reduced placental and fetal body volumes in CHD fetuses, with an elevated fetal-to-placental volume ratio, implying disproportionate placental underdevelopment [25]. Importantly, these alterations occurred regardless of lesion type, although certain phenotypes, like RSOl, revealed more severe abnormalities [24]. Functional imaging also revealed reduced placental pBOLD spatiotemporal variance in CHD cases, indicating disrupted vascularization and microstructure [23].

These imaging markers were complemented by Doppler ultrasound findings indicating elevated umbilical artery pulsatility index and decreased UVVF adjusted to fetal weight and as a proportion of fetal CCO (CPR) [25,26], suggestive of increased placental vascular resistance in pregnancies affected with CHD. Despite preserved absolute placental flow, CHD fetuses exhibit impaired weight-adjusted perfusion and altered vascular resistance patterns, suggesting that there may be some early placental dysfunction in this population.

Together, these studies suggest some degree of dysfunction of CHD-affected fetuses’ placentas regarding impaired perfusion, structural immaturity, and altered oxygenation patterns, all of which may contribute to suboptimal fetal growth and brain development.

### 4.2. Older Literature

A possible link between CHD and placental malfunction was already shown by several histology and ultrasound studies prior to the widespread adoption of MRI techniques for fetal screening. These early studies established that CHD may be a component of a larger maternal–fetal–placental circulatory and developmental pathology, rather than just a heart problem.

#### 4.2.1. Histopathology

The idea of placental dysfunction is supported by histopathologic examinations on placentas from fetuses affected by CHD, although these investigations have important limitations. These studies produce conflicting results because of the variability in completing placental pathologic evaluation in fetal CHD and the scarcity of some CHD diagnoses that require heterogeneous classification of CHD subcategories.

Placentas of pregnancies with CHD have a greater prevalence of thrombotic lesions, infarctions, and maternal vascular malperfusion (MVM), according to several placental pathology studies published in the 2000s and 2010s [29]. When Jones et al. examined placental histopathology in a group of newborns with HLHS, they discovered that the placenta had a higher frequency of infarcts, fibrin deposition, and reduced villous vascularity than controls [30]. These findings suggested chronic underperfusion and disruption of villous maturation as possible contributors to fetal compromise.

According to other findings, like these by Albalawi et al., all types of prenatal CHD have a higher prevalence of improper cord insertion, and fetuses with TGA have the highest number of placental anomalies [14].

In their report on placental pathology in 120 cases of CHD, Rychik et al. grouped similar heart lesions for subgroup analysis. They found that 41% of placentas in the whole CHD group had thrombosis, 17% had infarction, 18% had chorangiosis, and 15% had immature villi [16]. Notably, there was no control group in this study.

#### 4.2.2. Doppler/Magnetic Resonance Imaging

The current generation of fetal MRI studies builds directly on this foundation, offering in vivo proof of the same processes (impaired perfusion, disrupted vascular architecture, and chronic hypoxia) through quantitative and qualitative imaging markers [31]. On the same track, You et al. employed blood oxygen-level-dependent MRI (BOLD MRI) to demonstrate differential alterations in the fetal brain and placental BOLD signal with maternal hyperoxygenation in a cohort of pregnant women whose fetuses had been diagnosed with either biventricular or single-ventricle physiological CHD. The placenta of babies with single-ventricular physiology CHD showed a noticeably higher rise in BOLD signal than that of controls and fetuses with biventricular physiology CHD [32]. Likewise, fetuses with severe types of CHD demonstrate lower oxygen saturation in their umbilical vein than control groups, whereas their brain volume is reduced by 13% overall, according to research by Sun et al. using phase–contrast MRI and T2 mapping. These advances have the potential to contribute to earlier detection longitudinal tracking, but their role in the calculation of CHD risk remains to be clarified through further research [33].

Collectively, older studies consistently documented structural and vascular abnormalities in the placentas of fetuses with CHD. Although these studies lacked the spatial and temporal resolution provided by contemporary imaging, their histological and Doppler findings laid crucial groundwork. Combined with recent studies included in this review, they set the evidence that placental dysfunction is neither incidental nor secondary but may be an integral component of CHD pathophysiology.

### 4.3. Pathophysiological Considerations

The placenta being not only a passive supplier but also an active circulation regulator heavily influenced by the fetal heart, with the heart–placental axis also contributing to general fetal development, is an increasingly supported concept, though still under investigation [16,30,31]. But how exactly CHD leads to placental dysfunction is still under discussion.

#### 4.3.1. Hemodynamics

According to one of the most widely accepted theories, placental perfusion is hindered by abnormal fetal hemodynamics brought on by heart anomalies. Because the placenta and fetal heart form a closed circuit in pregnancy, modifications to the fetal heart’s output or blood flow distribution may have an impact on the dynamics of placental perfusion. This was demonstrated by the results of Josowitz et al., who found that although the absolute flow levels of CHD fetuses were similar, the indexed umbilical venous flow was considerably lower [20]. Localized hypoxia, villous maldevelopment, and disturbed angiogenesis can result from decreased perfusion efficiency on a kilogram-by-kilogram basis.

#### 4.3.2. Maternal Vascular Malperfusion (MVM)

Placental histopathologic abnormalities consistent with maternal vascular malperfusion (MVM)—such as diffuse signal loss and lobular heterogeneity on MRI—have been observed in CHD pregnancies (Steinweg et al.). However, MVM is a nonspecific lesion commonly seen in a range of obstetric conditions, including preeclampsia, hypertensive disorders, and fetal growth restriction, and cannot be uniquely attributed to CHD-induced placental pathology. Linask et al. described aberrant spiral artery remodeling and impaired trophoblast invasion—mechanisms shared by preeclampsia and FGR—which may similarly contribute to placental pathology in CHD [34].

In the context of CHD, malperfusion affects both maternal and fetal circulation, compounded by fetal polycythemia and hyperviscosity secondary to hypoxia, which elevates the risk of placental infarction. This leads to increased fibrin deposition, thrombosis, and large infarcts, impairing oxygen and nutrient transfer. Such changes may be worsened by compromised fetal cardiac output and hemodynamic stasis. Fetal imaging studies suggest placenta-mediated hypoxic abnormalities in CHD pregnancies [16,30,33].

Furthermore, maternal cardiovascular health has a significant impact on this condition. According to Rajagopalan et al., maternal risk factors such as diabetes and hypertension independently decrease BOLD signal variance, which makes fetal hypoxia in pregnancies affected by CHD even worse [23].

#### 4.3.3. Developmental Origins

Since the fetal heart and placenta are two of the first organs to differentiate, it is believed that their development is connected [35]. Abnormalities of the heart and placenta often co-occur due to polymorphisms in genetic developmental pathways that are active in both of these organs, particularly those controlled by Wnt/ß-catenin/planar cell polarity signaling, or due to a deficiency of essential micronutrients [34]. Basic functions of the placenta, such as cell adhesion and angiogenesis, are shared with the heart because, during evolution, the placenta co-opted gene networks originally involved in the development of other organs [36]. Thus, severe placental and cardiac defects are linked to the disruption of integrin alpha 4 or its ligand, the vascular cell adhesion molecule (VCAM-1).

While the epicardium and coronary arteries develop abnormally in the heart, the union of the allantois with the chorion fails in the placenta [37]. Similar circumstances occur with the conditional deletion of Hand1 in trophoblast progenitor cells when mouse embryos experience placental failure and fetal demise after embryonic day 10.5 due to defective labyrinth formation and inability to switch to hemotrophic nutrition [38]. Further mechanistic understanding of this gene is being researched in human subjects [39].

#### 4.3.4. Clinical Indicators for Placental Dysfunction

Traditional clinical indicators like gestational age at delivery and birth weight, including percentile-adjusted values, are still important indicators of placental dysfunction in pregnancies complicated by fetal CHD. These indicators provide context beyond the results of advanced imaging tests. Preterm (<39 weeks) and very preterm (<34 weeks) delivery risks are significantly higher for fetuses with CHD, according to multiple cohorts, and they are also consistently reported to be born earlier and with lower birth weights than those without CHD (e.g., 2835 vs. 3114 g on average) [40]. This is especially true for subtypes like tetralogy of Fallot, double-outlet right ventricle, and ventricular septal defects [27]. Notably, PW:BW ratios are frequently low in neonates with CHD, indicating increased placental efficiency despite small placental size, even though these neonates maintain relatively preserved birth weight and head circumference percentiles (median ~33rd and 35th) [41]. These clinical findings are supported by in vivo 3D MRI studies, which show reduced fetal body volumes and increased fetal-to-placental volume ratios in pregnancies with CHD, which suggests compromised placental support in relation to fetal growth [22]. When combined, these straightforward clinical endpoints—gestational age and birth weight centiles—provide essential, easily accessible markers of placental dysfunction and ought to be specifically included in studies and analyses of sophisticated imaging results in fetuses with congenital heart defects.

### 4.4. Limitations and Methodological Considerations

Despite their strengths, the included studies have some limitations:

Sample size and diversity: most of the studies had a small number of subjects (e.g., Cromb et al., CHD *n* = 12), limiting their statistical significance power. Moreover, CHD is a heterogeneous entity. Some studies categorized findings by lesion type, while others treated CHD as a single condition, potentially masking anomaly-specific results.

Imaging limitations: recent studies do not use a plethora of the available gestation imaging techniques, so this review is limited to MRI and Doppler study findings. While these methods, like BOLD and T2* techniques, offer great results, they are also susceptible to signal variation and artifacts, especially in late gestation [42]. There is emerging use of echo plantar imaging (EPI), a very promising tool [28].

Lack of longitudinal studies: Most studies were cross-sectional. There is still a need for more serial imaging studies with multiple follow-ups in order to understand the cooperative development and, thus, the implications of an abnormal fetal heart and the placenta.

Comorbidities and maternal risk factors: Maternal comorbidities, genetic syndromes, other fetal implications, and even more benign factors like fetal sex and gestational age can all influence the heart–placenta axis. While most studies adjusted their results for several basic factors like maternal and gestational age, only a few of them accounted for maternal and fetal environmental factors.

While mounting evidence indicates a correlation between placental structural or functional anomalies and CHD, contemporary studies are unable to ascertain the directionality or causality of this association. There are known shared developmental pathways between the placenta and fetal heart, especially those related to angiogenic signaling and early embryonic hemodynamics [43]. Nonetheless, the question of whether CHD independently deteriorates placental function, whether placental dysfunction contributes to CHD, or whether both arise from upstream developmental disturbances remains unanswered. Additionally, inadequate placental function is a key to various obstetric complications, including preeclampsia, fetal growth restriction, and placental insufficiency, which may coexist with or complicate associations with CHD.

One should not undervalue or ignore the possible impact of maternal comorbidities (such as diabetes and hypertension), intrauterine infection, and epigenetic changes that impact placental and cardiac morphogenesis. To separate this intricate relationship, longitudinal, mechanistic studies that account for these factors are crucial.

### 4.5. Future Research Directions

Future studies should aim to include larger, multicenter cohorts stratified by placental lesion type and maternal health status to enhance the generalizability of findings. Longitudinal MRI and Doppler imaging should be employed to monitor placental changes from early gestation, offering exciting perspectives on disease progression. Furthermore, imaging biomarkers must be correlated with placental histopathology and neonatal outcomes to validate their clinical relevance. Finally, randomized trials exploring therapeutic interventions, such as maternal oxygen therapy, are essential to assess their efficacy in improving placental function and fetal outcomes.

## 5. Conclusions

This review highlights a compact compilation of the most recent data there is, linking CHD to measurable abnormalities in the placenta, collected through imaging methods. These abnormalities, affecting placental structure, oxygenation, vascularization, and circulation, appear to be multifactorial and rooted in shared developmental pathways of the heart–placenta axis, dysregulated fetal hemodynamics, and maternal circulation factors. Modern imaging techniques, like fetal MRI and Doppler ultrasound, allow us to quantify and evaluate placental function compromise in utero, providing valuable insight into fetal well-being. Future research into the role of placental assessment in CHD-affected pregnancies is needed to clarify its potential in improving outcomes for these fetuses.

## Figures and Tables

**Table 1:** Risk of bias assessment for the included studies using the Newcastle–Ottawa Scale.

Study	Selection	Comparability	Outcome	Total Score
Cromb et al. [1]	3	1	2	6
Cromb et al. [10]	2	1	2	5
Jacobwitz et al. [22]	2	1	2	5
Rajagopalan et al. [23]	3	1	2	6
Steinweg et al. [24]	3	1	2	6
Josowitz et al. [25]	3	1	2	6
Ordas et al. [26]	3	1	2	6

**Table 2:** Characteristics of included studies.

Authors	Year	Study Design	Number of Participants (*n*)	Gestational Age at Scan	Congenital Heart Defects	Type of Image Study	Results
Cromb et al. [1]	2024	Prospective	CHD: 12 Controls: 36	CHD: 28.7–34.0 Controls: 26.9–32.6	CoA ToF TGA HLHS TA	Combined diffusion-relaxation MRI	Subjects with CHD had a significantly lower mean T2*. Volume and mean ADC (Apparent Diffusion Coefficient) values did not significantly differ across groups.
Cromb et al. [10]	2025	Retrospective	CHD: 51 Controls: 30	CHD: 30.9–32.9 Controls: 31.9–36.7	CoA TGA HLHS TOF CAT IAA AS APV MA PS DORV TAPVD AVSD	T2*-Relaxometry MRI	While placental volume and maximal placental thickness did not substantially change between groups, the CHD group’s placental T2* value and placental texture did. The CHD group’s placental morphology was noticeably better.
Jacobwitz et al. [22]	2025	Retrospective	CHD: 53 Controls: 55	CHD: 32.16 (mean) Controls: 28.11 (mean)	d-TGA HLHS TOF HRH VSD Truncus arteriosus Truncus with IAA Ebstein’s anomaly CAVC DORV AA and VSD Other	MRI	Throughout gestation, the CHD cohort’s placental volumes were noticeably lower than those of the control cohort.
Rajagopalan et al. [23]	2022	Prospective	CHD: 58 Controls: 114	CHD: 33.62 (mean) Controls: 32.27 (mean)	HLHS TGA ToF DORV TA, PA CoA AVC VSD ASD	MRI (pBOLD)	The inherent spatiotemporal pBOLD signal variance did not significantly differ between the CHD and non-CHD groups. The association between gestational age and intrinsic pBOLD spatial variance showed a negative interaction with CHD status, while the association between gestational age and temporal variance showed a significant interaction with CHD status.
Steinweg et al. [24]	2021	Prospective cross-sectional observational study	CHD: 69 Controls: 37	CHD: 31.3 (mean) Controls: 31.2 (mean)	HLHS CoA RSOL TGA VR Other	MRI	The placenta as a whole showed short T2* values, with extra and quicker deterioration from the lobules’ center to their perimeter. In RSOL, there was an increase in heterogeneity. In comparison to our control cohort, the CHD cohort seemed to exhibit advanced lobularity, increased granularity within the lobules at a given GA, and generally lower signal intensity across the placenta.
Josowitz et al. [25]	2025	Prospective	CHD: 38 Controls: 36	CHD: 22.3–26.8 Controls: 20.02–22.0	SV ToF d-TGA	US/ Doppler	There was no difference in absolute UVVF between patients and controls; however, in comparison to controls, UVVF was considerably lower in all cases and the SV subgroup when it was indexed to fetal weight (UVVF/Wt). When comparing cases to controls, the mean UA pulsatility index (UAPI) was higher and the mean uterine artery PI (UtAPI) was lower. There was no difference between CPR and middle cerebral artery PI (MCAPI).
Ordás et al. [26]	2022	Prospective	CHD: 71 Controls: 1773	CHD: 33.27 ± 5.512 Controls: N/A	Subgroup I: TGA, HLHS, AoAD Subgroup II: TOF, VSD, DORV, AVSD, TA, TRUN, PS, L-TGA	US/ Doppler	Higher UtA-PI in CHD-affected subjects than controls

* “observed” or “effective” T2.

## Data Availability

No new data were created or analyzed in this study.

## Supplementary Materials

The following supporting information can be downloaded at: https://www.mdpi.com/article/10.3390/diagnostics15172159/s1, Table S1: Search queries for literature search in different databases.
