# Supplementary material for: Unmasking the Placenta–Heart Axis: A Comprehensive Review of Placental Abnormalities in Congenital Heart Disease
Source: Diagnostics (Basel). 2025 Aug 26;15(17):2159. doi: 10.3390/diagnostics15172159 (PMC12428438; doi:10.3390/diagnostics15172159)
Supplement: Supplementary file 1 [file diagnostics-15-02159-s001.zip › diagnostics-3801270-supplementary.pdf]

Supplementary Table S1. Search queries for literature search in different databases.

PubMed/Medline	(("congenital heart disease"[MeSH Terms] OR "congenital heart defects"[MeSH Terms] OR "fetal heart defects"[Title/Abstract] OR "congenital heart disease"[Title/Abstract] OR "congenital heart defects"[Title/Abstract])<br>AND<br>("placenta"[MeSH Terms] OR "placental imaging"[Title/Abstract] OR "placenta insufficiency"[Title/Abstract] OR "placental vascularization"[Title/Abstract] OR "malperfusion"[Title/Abstract])<br>AND<br>("Doppler ultrasonography"[MeSH Terms] OR "magnetic resonance imaging"[MeSH Terms] OR "ultrasound"[MeSH Terms] OR "Doppler"[Title/Abstract] OR "ultrasound"[Title/Abstract] OR "MRI"[Title/Abstract] OR "pBOLD"[Title/Abstract] OR "vascularization"[Title/Abstract] OR "malperfusion"[Title/Abstract]))
Embase	('congenital heart disease'/exp OR 'congenital heart defect'/exp OR 'fetal heart defect':ti,ab OR 'congenital heart disease':ti,ab OR 'congenital heart defects':ti,ab)<br>AND<br>('placenta'/exp OR 'placental imaging':ti,ab OR 'placenta insufficiency':ti,ab OR 'placental vascularization':ti,ab OR malperfusion:ti,ab)<br>AND<br>('Doppler ultrasound'/exp OR 'ultrasound'/exp OR 'magnetic resonance imaging'/exp OR doppler:ti,ab OR ultrasound:ti,ab OR mri:ti,ab OR pBOLD:ti,ab OR vascularization:ti,ab OR malperfusion:ti,ab)
Scopus	(TITLE-ABS("congenital heart disease") OR TITLE-ABS("congenital heart defects") OR TITLE-ABS("fetal heart defects"))<br>AND<br>(TITLE-ABS(placenta) OR TITLE-ABS("placental imaging") OR TITLE-ABS("placenta insufficiency") OR TITLE-ABS("placental vascularization") OR TITLE-ABS(malperfusion))<br>AND<br>(TITLE-ABS(Doppler) OR TITLE-ABS(ultrasound) OR TITLE-ABS(MRI) OR TITLE-ABS("magnetic resonance imaging") OR TITLE-ABS(pBOLD) OR TITLE-ABS(vascularization) OR TITLE-ABS(malperfusion))
Web of Science	TS=("congenital heart disease" OR "congenital heart defects" OR "fetal heart defects")<br>AND

	TS=(placenta OR "placental imaging" OR "placenta insufficiency" OR "placental vascularization" OR malperfusion)<br>AND<br>TS=("Doppler" OR "ultrasound" OR "MRI" OR "magnetic resonance imaging" OR "pBOLD" OR "vascularization" OR "malperfusion")
Cochrane Library	("congenital heart disease" OR "congenital heart defects" OR "fetal heart defects")<br>AND<br>(placenta OR "placental imaging" OR "placenta insufficiency" OR "placental vascularization" OR malperfusion)<br>AND<br>(Doppler OR ultrasound OR MRI OR "magnetic resonance imaging" OR pBOLD OR vascularization OR malperfusion)
ClinicalTrials.gov	congenital heart disease OR congenital heart defects OR fetal heart defects)<br>AND<br>(placenta OR placental imaging OR placenta insufficiency OR placental vascularization OR malperfusion)<br>AND<br>(Doppler OR ultrasound OR MRI OR magnetic resonance imaging OR pBOLD OR vascularization OR malperfusion)
